# A randomized controlled trial of standard versus intensified tuberculosis diagnostics on treatment decisions by physicians in Northern Tanzania

**DOI:** 10.1186/1471-2334-14-89

**Published:** 2014-02-20

**Authors:** Elizabeth A Reddy, Boniface N Njau, Susan C Morpeth, Kathryn E Lancaster, Alison C Tribble, Venance P Maro, Levina J Msuya, Anne B Morrissey, Gibson S Kibiki, Nathan M Thielman, Coleen K Cunningham, Werner Schimana, John F Shao, Shein-Chung Chow, Jason E Stout, John A Crump, John A Bartlett, Carol D Hamilton

**Affiliations:** 1 Duke University Medical Center, Durham, NC, USA; 2 Kilimanjaro Christian Medical Centre, Box 3010, CCFCC Duke Projects, Moshi, Tanzania; 3 Kilimanjaro Christian Medical University College, Moshi, Tanzania; 4 KEMRI-Wellcome Trust Research Programme, Kilifi, Kenya; 5 Department of Epidemiology, University of North Carolina, Chapel Hill, NC, USA; 6 Children’s Hospital of Philadelphia, Philadelphia, PA, USA; 7 Duke Global Health Institute, Duke University, Durham, NC, USA; 8 Elizabeth Glaser Pediatric AIDS Foundation Tanzania, Dar Es Salaam, Tanzania; 9 Duke Clinical Research Institute, Duke University, Durham, NC, USA; 10 FHI 360, Durham, NC, USA

**Keywords:** Mycobacterium tuberculosis, Diagnosis, Health resources, Sputum/microbiology, HIV, adult, Child

## Abstract

**Background:**

Routine tuberculosis culture remains unavailable in many high-burden areas, including Tanzania. This study sought to determine the impact of providing mycobacterial culture results over standard of care [unconcentrated acid-fast (AFB) smears] on management of persons with suspected tuberculosis.

**Methods:**

Adults and children with suspected tuberculosis were randomized to standard (direct AFB smear only) or intensified (concentrated AFB smear and tuberculosis culture) diagnostics and followed for 8 weeks. The primary endpoint was appropriate treatment (i.e. antituberculosis therapy for those with tuberculosis, no antituberculous therapy for those without tuberculosis).

**Results:**

Seventy participants were randomized to standard (n = 37, 53%) or intensive (n = 33, 47%) diagnostics. At 8 weeks, 100% (n = 22) of participants in follow up randomized to intensive diagnostics were receiving appropriate care, vs. 22 (88%) of 25 participants randomized to standard diagnostics (p = 0.14). Overall, 18 (26%) participants died; antituberculosis therapy was associated with lower mortality (9% who received antiuberculosis treatment died vs. 26% who did not, p = 0.04).

**Conclusions:**

Under field conditions in a high burden setting, the impact of intensified diagnostics was blunted by high early mortality. Enhanced availability of rapid diagnostics must be linked to earlier access to care for outcomes to improve.

## Background

Tuberculosis remains a major worldwide public health problem, and difficulties in tuberculosis (TB) diagnosis have stalled progress in reducing the burden of disease, especially in endemic areas [1-3]. Tanzania ranks 18th among the 22 countries with the highest burden of TB worldwide [1], with an estimated incidence of TB of 312 cases/100,000 persons/year in 2007 at the time this research was initiated [4]. Unconcentrated sputum smear and symptomatology for smear-negative patients remain the mainstay of diagnosis [1,3,5-10], with bacterial culture limited by cost, infrastructure, and availability of trained personnel [11-13].

While availability of culture is assumed to improve management of tuberculosis in resource-limited settings, the degree to which availability of tuberculosis culture results change management decisions and clinical outcomes in a setting where clinical diagnosis is the norm has never been documented systematically. Early death [14-16], loss to follow up after initial sputum testing [14,17], and clinicians’ reluctance to exclude a diagnosis of tuberculosis even with a negative culture all may lessen the impact of tuberculosis culture results [18]. Carefully documenting the outcomes of patients followed with traditional (smear only) versus intensified (concentrated smear plus culture) diagnostic strategies would offer important information about prioritization and optimal implementation of tuberculosis diagnostics.

To address these questions, we conducted a randomized trial in Northern Tanzania of standard (acid fast bacilli (AFB) smear of gastric aspirate (GA) or sputum alone) or intensified (concentrated AFB smear and culture) diagnostics.

## Methods

### Study design and setting

This was a non-blinded individually randomized clinical trial. Subjects were recruited between November 2008 and July 2009 from Kilimanjaro Christian Medical Centre (KCMC), a 458 bed referral hospital for northern Tanzania, and Mawenzi Regional Hospital, a 300 bed regional hospital; both hospitals are located in Moshi, population ≈ 144,000 [19]. All inpatients were screened for inclusion; outpatients could also be referred to the study.

### Inclusion criteria

Adults (defined for the purposes of analysis as ≥6 years of age based on the unique characteristics of TB in younger versus older children and adults) and children (<6 years) were eligible for inclusion if they resided in the Kilimanjaro region with no plans to move during the study and consented to HIV testing. All potential subjects had to have 2 weeks of cough (and/or difficulty breathing for children) plus at least one of the following: recurrent fever, weight loss, night sweats, hemoptysis, failure to thrive (for children only), household contact with known/suspected tuberculosis (for children only), or a chest radiograph suspicious for tuberculosis. In addition, adult participants had to be able to provide at least one sputum specimen for testing.

### Study procedures

All patients (for participants ≥18 years of age) or their parent or guardian (for participants <18 years of age) provided written informed consent; assent was also required for participants aged 12 and older. Thereafter, a research questionnaire was completed and blood was drawn for HIV-1 antibody testing. CD4-positive T-lymphocyte cell count (CD4 count) and CD4 percentage were performed in the case of HIV-1 antibody positivity and HIV-1 antibody positivity was confirmed with HIV RNA in children ≤18 months of age. One sputum specimen was collected from all patients able to expectorate at the time of enrollment, with subsequent specimens collected the following 2 mornings for hospitalized patients or the following morning for outpatients. Therefore, a total of 3 samples were collected from hospitalized adults and a total of 2 samples were collected from outpatients; this was because of transportation challenges in requiring outpatients to return specimens. Children < 6 years of age who were not able to expectorate were hospitalized and 3 fasting GA samples were collected into specimen containers prefilled with 100 mg of sodium bicarbonate.

Tuberculin skin tests (TST; Mantoux method using 5 TU of tuberculin) were placed by trained study personnel and read 48–72 hours later, with 5 mm of induration considered reactive for HIV-infected participants and 10 mm for HIV uninfected participants. All participants were evaluated by posterior-anterior chest radiograph with results detailed onto standardized case report forms by an experienced radiologist. Treatment and diagnosis data were abstracted from hospital records.

Study-related test results were communicated verbally and in writing to treating clinicians. HIV-infected participants were referred to HIV Care and Treatment Centers (CTC) either at KCMC Hospital, Mawenzi Regional Hospital, or the CTC nearest to them. Outpatients, patients fit for hospital discharge, and pediatric patients with positive AFB smears and/or tuberculosis cultures were referred to the National Tuberculosis Control Program, located at Mawenzi Regional Hospital; but smear-positive patients requiring ongoing hospitalization were referred to Kibong’oto National Tuberculosis Hospital. The management of patients with TB was the same regardless of site of enrollment, however the follow up and management of patients without confirmed TB was at the discretion of treating clincians. Participants were seen for study visits 2 and 8 weeks after enrollment; those who did not attend appointments were traced by a field worker who came to their homes, at which point vital status and tuberculosis treatment status were ascertained. If no contact was made by 12 weeks post enrollment into the study, the participant was considered lost to follow up.

### Randomization

Randomization was stratified by age (adult ≥6 vs. child <6) using simple randomization tables generated by http://www.randomization.com. Randomization assignments were pre-prepared in ordered, sealed envelopes to be opened at the time that the participant’s first sputum or GA sample arrived in the laboratory.

### Laboratory procedures

Unconcentrated sputum and GA specimens from all participants in both groups were first assessed by trained laboratory technicians utilizing the Kinyoun modification of the Ziehl-Neelson stain. Information about the patient’s clinical condition was not available to the laboratory staff. For only the participants randomized to intensified diagnostics, specimens were decontaminated and centrifuged for concentration. Concentrated specimens were then evaluated using both Kinyoun staining and auramine-rhodamine fluorochrome staining with fluorescence microscopy. The intensified diagnostics group also had all specimens inoculated into a bioMerieux BacT/ALERT 3D Microbial Detection System. *Mycobacterium tuberculosis* complex growth was confirmed with DNA probe (AccuProbe Culture Identification Test MTB and MAC kits, Gen-Probe Inc., San Diego, CA).

### Diagnosis of tuberculosis and correct treatment

Participants who had at least one sputum smear positive for acid-fast bacilli (concentrated or un-concentrated), or at least one sputum or GA culture positive for MTB, were considered to have were considered to have microbiologically confirmed tuberculosis.

Participants who did not have microbiologically confirmed tuberculosis were evaluated by a pediatric or adult clinical endpoint committee (CEC) according to their age group. Each committee had 2 Tanzanian clinicians and one (pediatrics committee) or 2 (adult committee) U.S. infectious disease specialists; all were experienced in TB diagnosis. Committee members (who were not involved in care of the participants) were provided with standardized, de-identified clinical and radiographic data, including chest radiograph images. Committee members independently characterized participants into 2 diagnostic categories (tuberculosis or not tuberculosis). Determination of TB status was made by majority opinion among the CEC members. Since there were 4 members in the adult committee, it was pre-determined that an even split among the committee members on any one patient’s status would result in random removal of all of the ratings of one of the committee members.

### Statistical analysis

We assumed that 80% of enrolled participants would truly have tuberculosis [20-27], that 50% would be HIV-infected [28,29], and that the sensitivity of un-concentrated AFB smear for pulmonary TB was 38% in HIV-uninfected persons and 29% in HIV-infected persons (respective sensitivities of 50% and 38% were assumed for concentrated smears). Assuming 90% of intensified arm participants and 65% of standard arm participants will have been correctly prescribed therapy by 8 weeks, these assumptions would result in correct therapy (either anti-TB in the case of TB or no anti-TB in the case of no TB) for 93% of participants in the intensified diagnostic arm vs. 73% of participants in the standard arm (e.g. a participants in the standard arm (e.g. a 25% relative increase). A sample size of 120 (60 participants in each arm) was planned in order to afford at least an 80% power to detect an absolute difference of 20% in appropriate therapy between the 2 groups with a one sided Fisher’s exact test at the level of 0.025 significance.

Data were entered utilizing an automated Verity^®^ Teleform^®^ data entry system (Verity, Pleasonton, CA) into a Microsoft Access 2003 database; missing values or those which lay outside of anticipated ranges were re-checked for quality and manually entered as appropriate.

Participants who had microbiologically or clinically confirmed TB were considered to be correctly treated if they had initiated multi-drug anti-TB therapy and continued through their last study visit. Those for whom TB had been excluded on both clinical and microbiologic grounds who were not receiving anti-TB therapy were also considered to be correctly treated. All others were considered to be incorrectly treated. The primary endpoint was correct treatment at 8 weeks for patients who were alive and had 8-week follow-up data. Pre-specified secondary analyses included an assessment of correct treatment among those with smear-negative, culture-positive TB at 2 weeks, impact of specimen concentration on sensitivity and specificity among adults and children, and differences between HIV-infected and uninfected participants. All other statistical testing was post hoc, and assessment of association with mortality was explored carefully based on the somewhat unexpected high proportion of mortality. P-values were calculated using one-sided Fisher’s exact tests, and were not adjusted for multiple comparisons. Survival was assessed using Kaplan-Meier curves, and between-group differences were assessed with the log-rank test.

### Ethical considerations

The study was approved by the Kilimanjaro Christian Medical College Ethical Committee, the Tanzania National Institutes for Medical Research Ethical Coordinating Committee, and the Duke University Medical Center Institutional Review Board. The study has been registered with the Pan African Clinical Trials Registry (http://www.pactr.org) with the registry number PACTR201402000765312.

## Results

**Figure 1:**
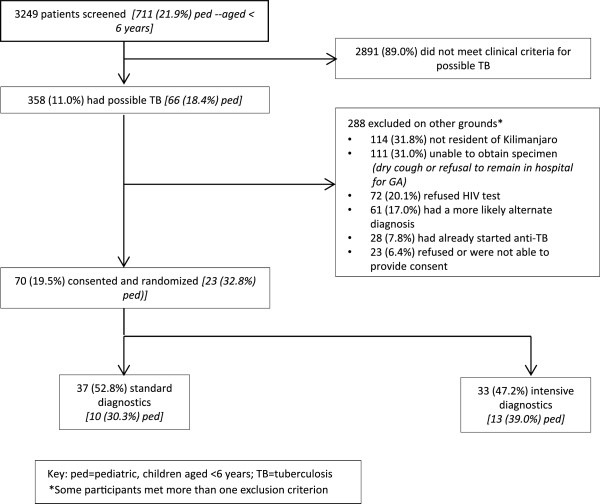
CONSORT diagram.

3,249 patients were screened for eligibility, 358 (11.2%) met clinical criteria for suspect tuberculosis, and 70 were enrolled (Figure 1 and Table 1). Enrollment was curtailed with the end of project funding, limiting the number of potentially eligible participants screened and enrolled.

**Table 1:** Baseline characteristics of 70 patients with suspect tuberculosis, Tanzania, 2008–9, by age and randomization group, n(%) or median (intraquartile range)

				**Age ≥6 years**		**Age <6 years**	
	***All***	***Standard***	***Intensified***	***Standard***	***Intensified***	***Standard***	***Intensified***
All	70	37	33	27 (57)	20 (43)	10 (43)	13 (57)
Female	35	21 (57)	14 (42)	14 (52)	9 (45)	7 (78)	5 (42)
Age (years), participants ≥6 years	37 (28–48)	37 (28–48)	37 (30–46)	38 (29–50)	38 (31–46)	n/a	n/a
Age (months), participants <6 years	16 (6–22)	21 (5–47)	11 (6–19)	n/a	n/a	21 (5–47)	10.5 (6–18)
Enrolled from outpatient	4 (6)	1 (3)	3 (9)	1 (4)	3 (15)	0	0
Days ill prior to enrollment	21 (16–60)	30 (20–60)	21 (14–44)	45 (30–60)	21 (14–60)	14 (12–17)	20 (14–36)
Fever	52 (74)	27 (73)	25 (76)	19 (70)	13 (65)	8 (80)	12 (92)
Cough	68 (97)	35 (95)	33 (100)	27 (100)	19 (95)	9 (90)	13 (100)
Weight loss	45 (64)	22 (60)	23 (70)	17 (63)	16 (80)	5 (50)	7 (54)
Night sweats	21 (30)	10 (27)	11 (33)	10 (37)	6 (20)	1 (10)	4 (31)
Clinical suspicion extrapulmonary TB	3 (4)	2 (5)	1 (3)	2 (7)	1 (5)	0	0
Chest radiograph suspect TB*†	31 (50)	19 (51)	12 (41)	15 (56)	9 (45)	3 (30)	4 (30)
Chest infiltrates, no other suspicious features	19 (31)	8 (24)	11 (38)	4 (15)	5 (20)	7 (60)	3 (23)
Previously treated for TB	4 (6)	3 (8)	1 (3)	3 (11)	1 (5)	0	0
Malnourished**	39 (56)	20 (54)	19 (58)	15 (56)	11 (55)	5 (50)	8 (62)
HIV infection	34 (49)	15 (41)	19 (58)	13 (48)	13 (65)	2 (20)	6 (46)
<12 months since HIV diagnosis†‡	28 (82)	15 (100)	13 (68)	8 (30)	13 (100)	1 (50)	5 (83)
Antiretroviral therapy at enrollment†	13 (38)	5 (33)	8 (42)	4 (15)	7 (35)	1 (50)	1 (17)
CD4+ cell count/mm3‡	180 (10–405)	172 (17–293)	180 (9–578)	172 (15–291)	19 (8–229)	n/a	n/a
CD4+ cell percentage‡	0.9 (0.2–16)	0.5 (0.2–21)	0.9 (0.2–16)	n/a	n/a	23 (0.5-45)	16 (8–30)

*Presence of cavitation, micronodules, pleural effusion with infiltrate, or infiltrate pattern suspicious for TB as per radiologist.

**Body mass index <20 or weight for height >2 standard deviations below mean.

†Percentages of those with available data.

‡Among HIV infected.

Sixty-five (92.9%) participants provided at least 2 sputum or GA specimens. Direct (unconcentrated) sputum or GA smears for AFB were positive in 11 (15.7%) (6 HIV-infected and 5 HIV-uninfected), and culture was positive for *Mycobacterium tuberculosis* in an additional 6 (18.2%) of 33 participants in the intensified arm, for a total of 17 (24.3%) participants with microbiologically confirmed tuberculosis. Concentrated smear detected AFB in 4 samples which were direct smear negative; the sensitivity of concentrated smear was 42.8% and of direct smear was 32.1%.

In addition to the 17 participants with positive AFB smears and/or TB cultures, 14 participants [4 (20.0%) children and 10 (29.4%) adults, p = 0.51] without a microbiological diagnosis of TB were diagnosed with clinical TB by the CEC. Therefore, a total of 31 (44.3%) participants had a final diagnosis of TB.

Forty-seven participants were alive and in follow up to be assessed for the primary endpoint of treatment at 8 weeks (Table 2). All patients in the intensive arm (100%; n = 22) were receiving correct care vs. 22 (88.0%) of the 25 participants in the standard arm (p = 0.14). In the standard arm all 9 patients determined to have clinical or microbiologically confirmed TB were receiving anti-TB medications but 3 (18.8%) of 16 patients determined not to have TB by the CEC were continuing anti-TB medications. All participants with smear positive TB who were alive at 8 weeks were correctly receiving anti-TB medications.

**Table 2:** Odds of correct care* by randomization group among 70 patients with suspect tuberculosis, Tanzania, 2008-9

	**Standard diagnostics**			**Intensified diagnostics**			**OR (CI)**	**p**
**Correct care at 8 weeks (for living participants in follow up; n = 47)**								
	n for group	n correct care	% correct care	n for group	n correct care	% correct care		
All participants	25	22	(88)	22	22	(100)	Undefined	0.14
TB+	9	9	(100)	9	9	(100)	1	
TB-	16	13	(81)	13	13	(100)	Undefined	0.15
Smear negative participants	20	17	(85)	19	19	(100)	Undefined	0.12
TB+	4	4	(100)	6	6	(100)	1	
TB-	16	13	(81)	13	13	(100)	Undefined	0.15
**Correct care censored at death or last study visit; n = 70**								
	n for group	n correct care	% correct care	n for group	n correct care	% correct care		
All participants	37	26	(70)	33	29	(88)	3.1 (0.87,10.8)	0.06
TB+	16	9	(56)	16	11	(69)	2.1 (0.47, 9.7)	0.27
TB-	21	17	(81)	18	18	(100)	Undefined	0.07
Smear negative participants	32	21	(66)	27	24	(89)	4.2 (1.0,17.1)	0.04
TB+	11	4	(36)	9	6	(67)	3.5 (0.55, 22.3)	0.18
TB-	21	17	(81)	18	18	(100)	Undefined	0.07

*Defined as TB therapy if designated to have clinically (by clinical endpoint committee) or microbiologically diagnosed TB, or no TB therapy if determined not to have TB.

Six participants were AFB smear negative and TB culture positive. While 4 (66.7%) of these were culture positive prior to the 2 week visit, 3 of the 4 died prior to or within 72 hours of receipt of culture results and were never started on anti-TB medications, and the other was empirically started on anti-TB medications prior to culture results. The remaining 2 participants, whose cultures became positive after the 2 week visit, were started on anti-TB medications after receipt of culture results.

In a post hoc analysis of all participants utilizing last available treatment status prior to death or loss to follow up, participants in the intensified arm were more likely to receive correct care than participants in the standard arm [29 (87.9%) of 33 vs. 26 (70.3%) of 37; OR 3.1, p = 0.06]. This trend was also noted among smear-negative participants (24 (88.9%) of 27 received correct treatment in the intensified arm vs. 21 (65.6%) of 32 in the standard arm; OR = 4.1, p = 0.04]. Among 4 participants in the intensified arm and 11 participants in the standard arm who were treated incorrectly, 12 were not receiving anti-TB medication when it would have been recommended by the CEC, and 3 were receiving anti-TB medication when it would not have been recommended.

In a post hoc analysis of mortality, 18 (25.7%) of the 70 participants died during the study period at a median of 14 (IQR 7.8-26) days after enrollment, and an additional 7 (10.0%) participants were lost to follow up. The median time to death was 13.5 days (IQR 8,24). All of the patients who died were initially enrolled in the inpatient setting. No direct association was detected between death and assignment to standard or intensive diagnostics, presence of smear positivity, age group, nor days of illness prior to enrollment. Mortality was also not significantly associated with HIV status among all patients; for patients with HIV, death was not associated with severity of immune suppression or use of antiretroviral therapy.

**Figure 2:**
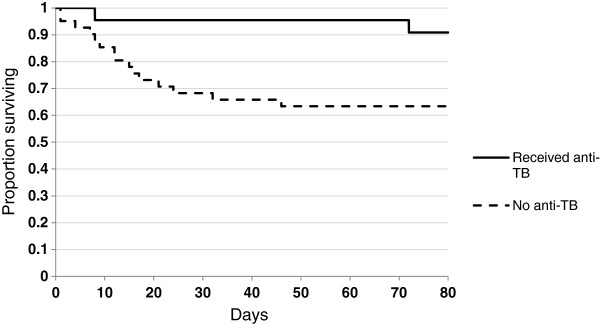
Survival by receipt of antituberculous treatment among 70 participants with suspect tuberculosis, regardless of randomization group, Tanzania, 2008–9.

Receipt of anti-TB medications was inversely associated with death (OR 0.2, p = 0.04; see Table 3, Figure 2) in the cohort as a whole and among HIV-infected participants and adults; HIV-infected participants who received anti-TB were significantly less likely to die than those who didn’t (OR undefined, p = 0.01) as were adults who received anti-TB compared with those who didn’t (OR 0.19, p = 0.02). Six (35.3%) of 17 patients with microbiologically confirmed TB died, 5 of whom never received anti-TB medications. Of these 5 who had microbiologically confirmed TB but never received medications, 4 died within 3 days of the first positive sputum or gastric aspirate specimen, and 1 died 10 days after return of the first positive gastric aspirate specimen. Four of the 5 were enrolled from KCMC, the tertiary referral hospital, where anti-TB is not available on-site. Of the 11 patients who died without a microbiologically confirmed diagnosis of TB and did not receive anti-TB, 9 died with a diagnosis of pneumonia and/or HIV/AIDS.

**Table 3:** Associations with death and receipt of antituberculous treatment among 70 participants with suspect tuberculosis Tanzania, 2008-9

	**All**	**Anti-TB**	**No anti-TB**	**OR**	**p**
**All**	70	22 (31)*†	48 (68)		
Death before 2 weeks	9 (13)	1 (4)	8 (17)	0.25 (0.03–2.0)	0.25
Any death	18 (26)	2 (9)	16 (33)	0.2 (0.04–0.96)	**0.04**
**Pediatric**	23	7 (30)	16 (69)		
Death before 2 weeks	2 (9)	1 (14)	1 (6)	2.5 (0.13–46.8)	0.53
Any death	4 (17)	1 (14)	3 (19)	0.72 (0.06–8.5)	0.66
**Adult**	47	15 (32)	32 (68)		
Death before 2 weeks	7 (15)	0	7 (22)	undefined	0.08
Any death	14 (30)	1 (7)	13 (41)	0.10 (0.01–0.89)	**0.02**
**HIV-infected**	34	10 (29)	24 (71)		
Death before 2 weeks	5 (15)	0	5 (21)	undefined	0.29
Any death	9 (26)	0	9 (38)	undefined	**0.01**
**Smear-negative**	59	13 (22)	46 (78)		
Death before 2 weeks	7 (12)	0	7 (15)	undefined	0.33
Any death	15 (25)	1 (8)	14 (30)	0.19 (0.02–1.6)	0.15
**Standard arm**	37	12 (32)	25 (68)		
Death before 2 weeks	4 (11)	0	4 (19)	undefined	0.19
Any death	9 (24)	1 (8)	8 (32)	0.19 (0.05–1.5)	0.12
**Intensified arm**	33	10 (30)	23 (70)		
Death before 2 weeks	5 (15)	1 (10)	4 (17)	0.53 (0.20–1.4)	0.29
Any death	9 (27)	1 (10)	8 (35)	0.20 (0.00–1.4)	0.15

*An additional 2 patients were prescribed anti–TB but died before initiating therapy.

†Percentages refer to proportion of total in each category.

p–values in bold are statistically significant at a level of p<0.05.

## Discussion

The impact of intensified microbiologic testing on TB management in our study was attenuated by a strikingly high mortality rate, even among HIV-negative subjects. More than one quarter of the study participants died prior to the end of the 8 week study period, including a third of the patients with microbiologically confirmed tuberculosis; this parallels or exceeds mortality seen in case series of patients with TB in other high-burden, HIV-prevalent settings [14-16,30]. Mortality was seen in all major subgroups, including HIV infected and uninfected participants and children as well as adults. Enrollment primarily from the wards of a tertiary referral hospital surely contributed to these findings, and they underscore the ongoing challenges for providing quality, accessible primary health care in a resource-limited setting. Five of those with confirmed TB died prior to initiation of anti-TB; in 4 of these 5 cases, results were available only after death or within 72 hours prior to death. Most importantly, these results suggest that better diagnostic testing at tertiary care centers in developing world settings are unlikely to change outcomes without measures to improve access to such testing among persons in the community, and/or community awareness of the importance of early presentation with symptoms possibly compatible with TB. While late diagnosis was likely the major contributor to mortality in this cohort, it is important to note that anti-TB medications are not always immediately available to clinicians in our setting. For example, anti-TB medications must be dispensed from the regional TB office adjacent to Mawenzi Regional Hospital during weekdays only. While it is not clear that earlier receipt of anti-TB by a few days in critically ill patients would have been life-saving, it may have been; means to ensure more rapid receipt of anti-TB in emergency cases have been considered and ongoing discussions are in progress.

Despite the overarching message of our data that TB needs to be considered and empiric therapy may save lives, overtreatment was noted in our study and formed the major difference between the standard and intensive groups in terms of the primary endpoint of correct treatment at 8 weeks. The lack of ability to obtain cultures and/or molecular diagnostics may result in both overtreatment as well as under treatment of TB, possibly leading the clinician away from consideration of other diagnoses and exposing patients to medication toxicity [31,32].

In addition, 10% of our cohort was lost to follow up despite obtaining contact information and attempts to reach them by a field worker. One-third of smear negative participants in the intensified arm were culture positive. While we worked closely with the National TB Control Program to contact the 2 culture-positive participants who were not already being treated, tracking down smear-negative, culture-positive persons after they have left initial care will require significant resources that may not be available, again underscoring the importance of rapid diagnostics.

Our study has several important limitations, including being underpowered due to a lengthy regulatory process and subsequent delays in enrollment, a substantial proportion of symptom-eligible patients excluded on other grounds, and the lack of a gold standard diagnosis. The latter was mitigated by the use of the CEC.

Despite these limitations, to our knowledge this is the first study to use a prospective, randomized approach to examine the clinical benefit of the addition of TB culture to smear microscopy alone in a resource limited settings, where to date implementation of culture to improve diagnosis remains slow.

## Conclusions

Our data suggest that access to more sensitive TB diagnostics is associated with more appropriate therapy, both for those with and without TB. The high mortality seen in our study, present across all age groups and among patients with and without HIV, points to the need for operational studies coupling earlier access to care with intensified diagnostics for TB suspects.

## Competing interests

All authors declare no conflicts of interest nor competing interests.

## Authors’ contributions

EAR, CH, and JES wrote the manuscript with significant contributions from CKC and WS. CH, JAB, JAC, GSK, LJM, ACT, CKC, JES and JFS contributed to the design of the study. BNN, KL, JAC, JAB, NMT, VPN, SCC, and EAR contributed to acquisition and analysis of data from hospital wards, and SCM and ABM to acquisition and analysis of data in the laboratory. All authors read and approved the final manuscript.

## Pre-publication history

The pre-publication history for this paper can be accessed here:

http://www.biomedcentral.com/1471-2334/14/89/prepub
